# Development and evaluation of a web-based capacity building course in the EUR-HUMAN project to support primary health care professionals in the provision of high-quality care for refugees and migrants

**DOI:** 10.1080/16549716.2018.1547080

**Published:** 2018-11-30

**Authors:** Elena Jirovsky, Kathryn Hoffmann, Elisabeth Anne-Sophie Mayrhuber, Enkeleint Aggelos Mechili, Agapi Angelaki, Dimitra Sifaki-Pistolla, Elena Petelos, Maria van den Muijsenbergh, Tessa van Loenen, Michel Dückers, László Róbert Kolozsvári, Imre Rurik, Danica Rotar Pavlič, Diana Castro Sandoval, Giulia Borgioli, Maria José Caldés Pinilla, Dean Ajduković, Pim De Graaf, Nadja van Ginneken, Christopher Dowrick, Christos Lionis

**Affiliations:** a Department of General Practice and Family Medicine, Medical University of Vienna, Vienna, Austria; b Clinic of Social and Family Medicine, School of Medicine, University of Crete, Heraklion, Greece; c Department of Primary and Community Care, Radboud University Medical Centre, Nijemegen, The Netherlands; d Netherlands Institute for Health Services Research; e Department of Family and Occupational Medicine, Faculty of Public Health, University of Debrecen, Debrecen, Hungary; f Department of Family Medicine, University of Ljubljana, Lubljana, Slovenia; g European Forum for Primary Care, Utrecht, The Netherlands; h Azienda USL Toscana Centro - Global Health Center, Region of Tuscany, Florence, Italy; i Centro Salute Globale, Tuscany; j Department of Psychology, Faculty of Humanities and Social Sciences, University of Zagreb, Zagreb, Croatia; k Primary Medical Care, University of Liverpool, Liverpool, United Kingdom

**Keywords:** Refugees, migrants, primary health care, capacity building, training

## Abstract

**Background**: The ongoing refugee crisis has revealed the need for enhancing primary health care (PHC) professionals’ skills and training.

**Objectives**: The aim was to strengthen PHC professionals in European countries in the provision of high-quality care for refugees and migrants by offering a concise modular training that was based on the needs of the refugees and PHC professionals as shown by prior research in the EUR-HUMAN project.

**Methods**: We developed, piloted, and evaluated an online capacity building course of 8 stand-alone modules containing information about acute health issues of refugees, legal issues, provider–patient communication and cultural aspects of health and illness, mental health, sexual and reproductive health, child health, chronic diseases, health promotion, and prevention. The English course template was translated into seven languages and adapted to the local contexts of six countries. Pre- and post-completion knowledge tests were administered to effectively assess the progress and knowledge increase of participants so as to issue CME certificates. An online evaluation survey post completion was used to assess the acceptability and practicability of the course from the participant perspective. These data were analyzed descriptively.

**Results**: A total of 390 participants registered for the online course in 6 countries with 175 completing all modules of the course, 47.7 % of them medical doctors. The mean time for completion was 10.77 hours. In total, 123 participants completed the online evaluation survey; the modules on acute health needs, legal issues (both 44.1%), and provider–patient communication/cultural issues (52.9%) were found particularly important for the daily practice. A majority expressed a will to promote the online course among their peers.

**Conclusion**: This course is a promising learning tool for PHC professionals and when relevant supportive conditions are met. The course has the potential to empower PHC professionals in their work with refugees and other migrants.

## Background

In 2015, the number of refugees from the Middle Eastern and Sub-Saharan countries entering European countries highly increased [1]. That year, close to 1.3 million people applied for asylum within the countries of the European Union [2]. The refugees arrived mainly to the Greek islands, and continued travelling through the Western Balkan route toward their destination countries in Northern Europe [3]. The migratory flow was halted in March 2016 due to more restrictive migration policies [4].

The population on the move and – at the point of and following arrival – in the destination countries was, and still is, in need of health care. European countries were very concerned about refugees bringing infectious diseases to their countries; however, evidence to corroborate these particular fears was missing [5]. Overall, the health problems of refugees and migrants are similar to those of the rest of the population ranging from accidental injuries, reproductive health issues, to chronic diseases. The latter constitute a particular problem, as care is very likely interrupted during and after migration [6–8]. Female refugees especially face challenges in regard to sexual and reproductive health, and might have been victims of violence prior or during the flight [6,9–11]. Furthermore, pre-migration physical or psychological traumas and post-arrival challenges, as well as prior mental health issues, can contribute toward the development of various mental health issues, often becoming manifest upon or after arrival in the destination countries [12].

Countries that have received and registered a large number of refugees, are struggling to meet the new population’s health care needs – particularly those countries that recently underwent a financial crisis and/or protracted austerity period like Greece [13,14]. The significant increase in the number of people in a relative short period of time combined with sparse resources caused various challenges particularly for primary health care (PHC) professionals in all destination countries of refugees, as PHC is the first point of entry to the health care system in most countries. There are many differences and similarities in the provision of health care services in different EU countries.

The European Refugees – Human Movement and Advisory Network (EUR-HUMAN project) (website: http://eur-human.uoc.gr/) was funded by the 3rd Health Program of the European Union (EU) and ran from January to December 2016. In this project, measures and interventions for an improvement of primary health care delivery for refugees and other migrants were identified, designed, assessed, and implemented. The objective was to reinforce and develop skills, abilities, and know-how in this field, particularly in EU-member states receiving refugees and other migrants. The health needs of these vulnerable groups were addressed so as to safeguard all population groups in EU-member states from specific health-related risk factors, and to minimize cross-border health risks.

Qualitative research conducted in the framework of the EUR-HUMAN project in seven EU countries (Austria, Croatia, Greece, Hungary, Italy, the Netherlands, and Slovenia) [15,16], showed challenges that concerned both the PHC professionals and the refugees and other migrants in need of health care: knowledge gaps, systemic challenges regarding social insurance and health insurance, language barriers, and communication differences were identified as particularly challenging [15,17]. On the one hand culture-related communication differences were identified as hampering for mental health diagnoses, on the other hand there is a lack of mental health care options available targeted especially to refugees [15,16]. The results of this research are consistent to earlier studies [18–20].

The above-mentioned reasons illustrate the need for strengthening PHC professionals so as to enable them to provide adequate health care to refugees and other migrants. The project EUR-HUMAN overall aimed to strengthen the knowledge and skills of PHC professionals in European countries involved in PHC for refugees, asylum seekers and other newly arrived migrants; second, it aimed to support the PHC professionals in European countries to provide high-quality primary care for refugees in an informed, integrated, person-centered, as well as competent and safe way (both for the refugee and the provider) [21].

It has been shown that e-learning in PHC can i.e. strongly enrich continuing medical education in this field [22]. Within the framework of the EUR-HUMAN project, the project team of the Medical University of Vienna (MedUni) developed a template in English for an online capacity building course. This course is a concise modular training that is based on the needs of the refugees and PHC professionals as assessed in the project.

## Methods

### Program development

**Figure 1.**
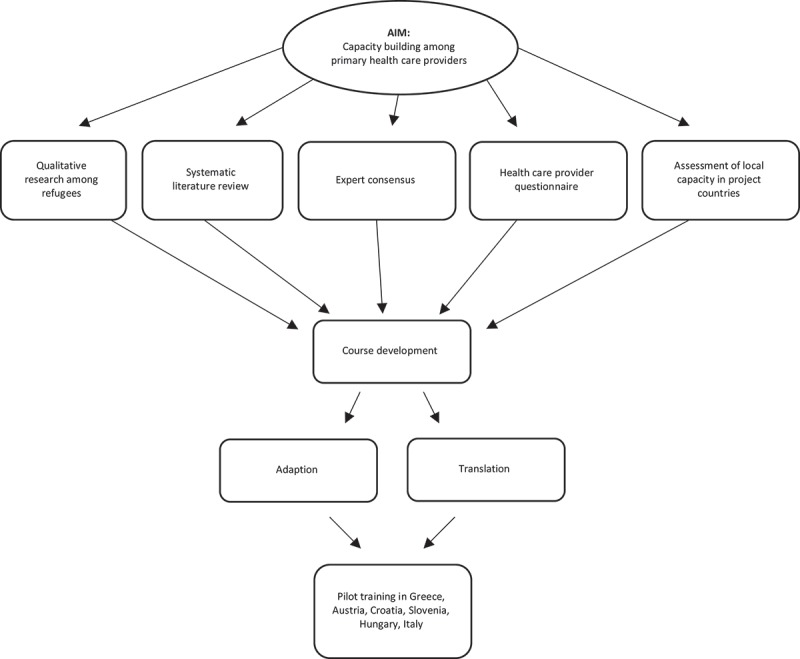
Flowchart CME course development.

The EUR-HUMAN online course was developed in the context of the EUR-HUMAN project and was piloted between October and December 2016 in six different EU countries (Greece, Hungary, Italy, Austria, Croatia, and Slovenia). It was one of the core interventions of the project. The online course was designed under the leadership of the MedUni in Work Package (WP 6) of the project, which had the aim to translate available knowledge and guidelines into capacity building training programs [21]. The design of the course was informed by results of the other WPs of the EUR-HUMAN project (see Figure 1): interviews with refugees (living in hotspots, transit centers and long-term stay centers) GPs, and other personnel involved across different organizational levels of PHC [15–17], as well as via an international systematic and narrative literature review [23] and an expert consensus meeting [24]. Furthermore, already existing materials from International Organization of Migration (IOM), European Center for Disease Control (ECDC), and previously conducted relevant projects, were included.

This online course aimed to support capacity building of PHC professionals. Its main target is to provide missing information regarding different issues in the context of PHC for refugees and other migrants in the destination countries. The web-based online course for PHC professionals was developed to include text, as well as audiovisual media (e.g. photos, videos, etc.) and links to relevant resources, including documentation and information in organizations providing refugee aid. The course was designed to provide information in an easily accessible form over a relatively short period of time, as the target group often faces a high workload [25]. The aim was to develop a training that was concise while still containing the most important information. The training takes approximately 11 hours learning hours and can be easily managed in addition to a full-time employment within 4 weeks.

The course followed a modular design. Multiple experts, both from the research team at MedUni (2 medical anthropologists, 1 PHC specialist, 1 medical student, and 1 vaccination expert) and external partners (2 pediatricians, 2 legal experts, a team of mental health specialists, 1 psychotherapist, a team of experts on women’s health issues, 1 expert from the Austrian Red Cross) created the content of the modules. First, a template version was developed in English. This consisted of eight modules, including an introductory one. Each module had several chapters covering the various topics relevant to the care for refugees and other migrants. The original template version in English can be used as basis to develop similar initiatives in case stakeholders or policymakers are interested in transferable practices, best practices, and available tools.

Apart from the language, the content of the template needs to be adapted to each country’s characteristics, as the legal systems, health care systems, epidemiology, as well as links to helpful organizations and information differ. Furthermore, the content needs to be modified depending on the target‐groups’ composition (physicians, nurses, midwifes, health visitors, PHC teams etc.).

For the pilot between October and December 2016, MedUni provided an adaptation and translation guideline to the partners together with the English template. For Austria, the entire English version was translated into German for Austrian PHC professionals and, (in a modified version) into Arabic for PHC professionals among Arabic speaking refugees who aspired a recognition of their certificates in Austria. This adaption comprised target group-specific revisions regarding legal regulations for volunteer work as asylum seeker and information on the process of validation of foreign study degrees in Austria.

The translation was partly done by members of the EUR-HUMAN teams (Croatian, German, Greek, Hungarian, Italian), and partly by official translation agencies (Arabic, Slovenian); and the content was adapted to the respective local needs regarding for instance the legal regulations in each country and the information on local refugee aid organizations.

The course was hosted by Health[e]Foundation, a Dutch organization specialized in trainings for health care workers (http://www.healthefoundation.eu/). Upon a self-explanatory and user-friendly online registration, participants received a login code and password. The course format allowed the target groups to do the training on any device, including mobile devices, in any chosen location, with individual time management. It was possible to alternate between modules and chapters.

The target group in Austria were GPs, as they are the main PHC professionals. In Croatia, the situation is similar: a large number of GPs deliver PHC services. Croatia is not a preferred destination country and PHC professionals are not experienced in providing services to migrants. In Italy, refugees and other migrants are enrolled in the National Health Service; the target group included GPs, nurses, and midwives. In Greece, different target groups included both PHC professionals and government officials, civil servants, and local stakeholders on the island of Lesvos. In Hungary, the target group consisted of PHC professionals experienced in working with migrants and refugees, or those interested in the knowledge conveyed in the course. In Slovenia, as well, PHC professionals experienced working with migrants and refugees where the target group. In Greece, Hungary, and Italy, face-to-face trainings were held before the participants started the online course, so as to facilitate the uptake of the online training.

### Program content

The program content was organized in eight stand-alone modules, including an introductory one, with multiple subsections: (1) Introduction; (2) Acute health needs of refugees; (3) Legal issues; (4) Provider–patient communication; (5) Mental health; (6) Sexual and reproductive health; (7) Child health; (8) Chronic disease, health promotion, and prevention (for a more detailed overview of the subsections of the modules see additional file 1 and http://eur-human.uoc.gr). The modules were designed for PHC professionals, who are involved in PHC for refugees and other newly arrived migrants. After registration, the user was directed to Module 1, the introductory module with instructions for the course. The online course incorporates audiovisual material like pictures, graphical representations, including statistical ones, excerpts from policy and guidance documentation, links to relevant resources in external websites, to videos, to external documents, etc., and to organizations providing refugee aid. These links need regular update to ensure that they remain up-to-date.

Module 1: Introduction.

Module 1 introduces the learner to the background and aims of the EUR-HUMAN project. Furthermore, basic instructions on the course are given and the theory behind the course is explained.

Module 2: Acute health needs of refugees.

**Figure 2.**
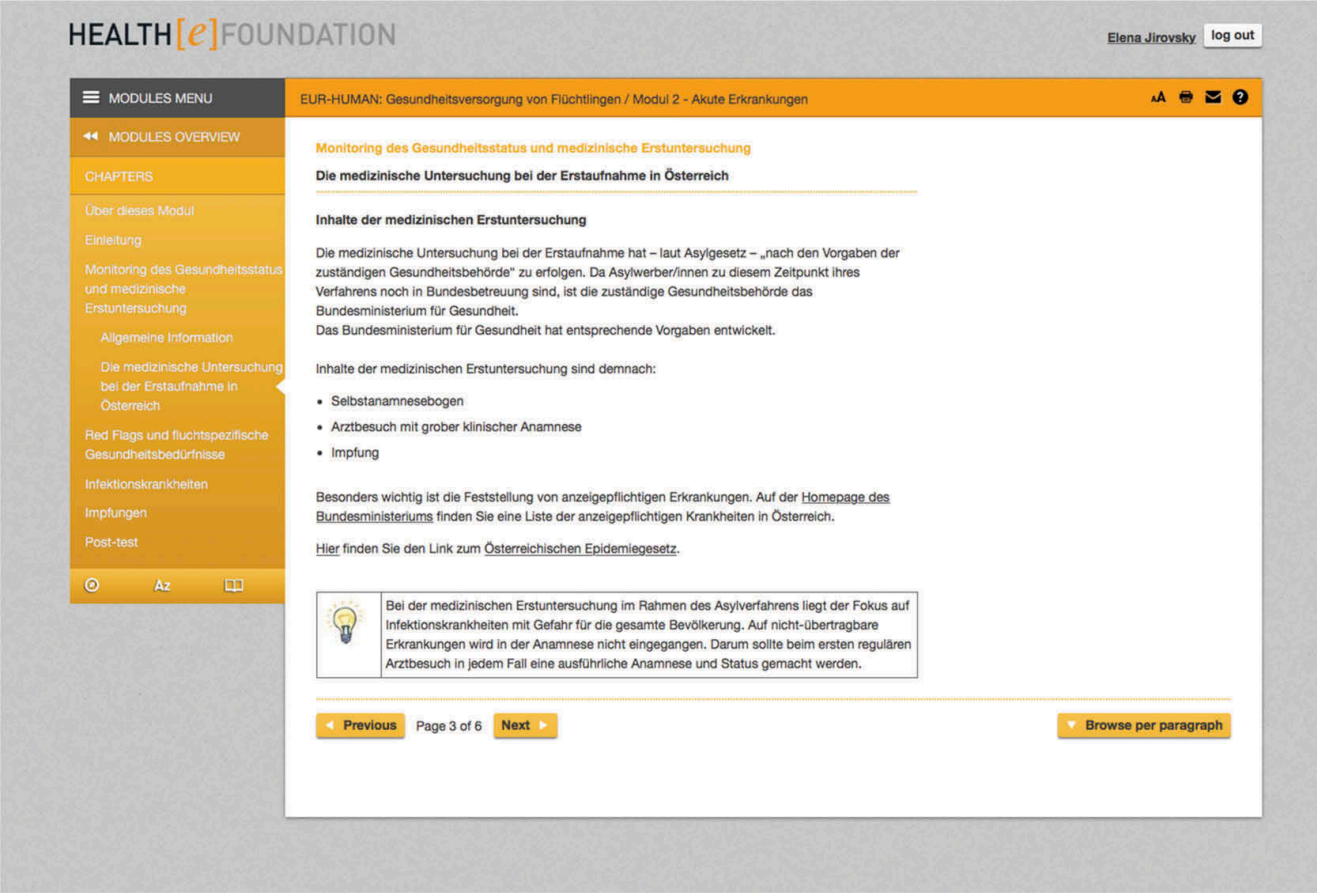
Module 2 – The initial health assessment for refugees in Austria (German).

Module 2 gives the learner an insight into health care-related processes upon arrival of the refugees and other migrants in a given country, during the registration procedure, before they enter the regular health care system. The module deals with various health care-related issues of newly arrived refugees and other migrants and highlights the need for a continuity of care between countries of origin, transit and destination. Flight-specific health needs and red flags in a short-stay setting, as well as infectious diseases, and vaccination coverage are discussed (see Figure 2).

Module 3: Legal issues.

Module 3 discusses legal questions regarding the medical care for refugees and other migrants during their asylum procedure and beyond. Probable solutions for the use of interpreters, translators and cultural mediators are portrayed.

Module 4: Provider–patient interaction.

**Figure 3.**
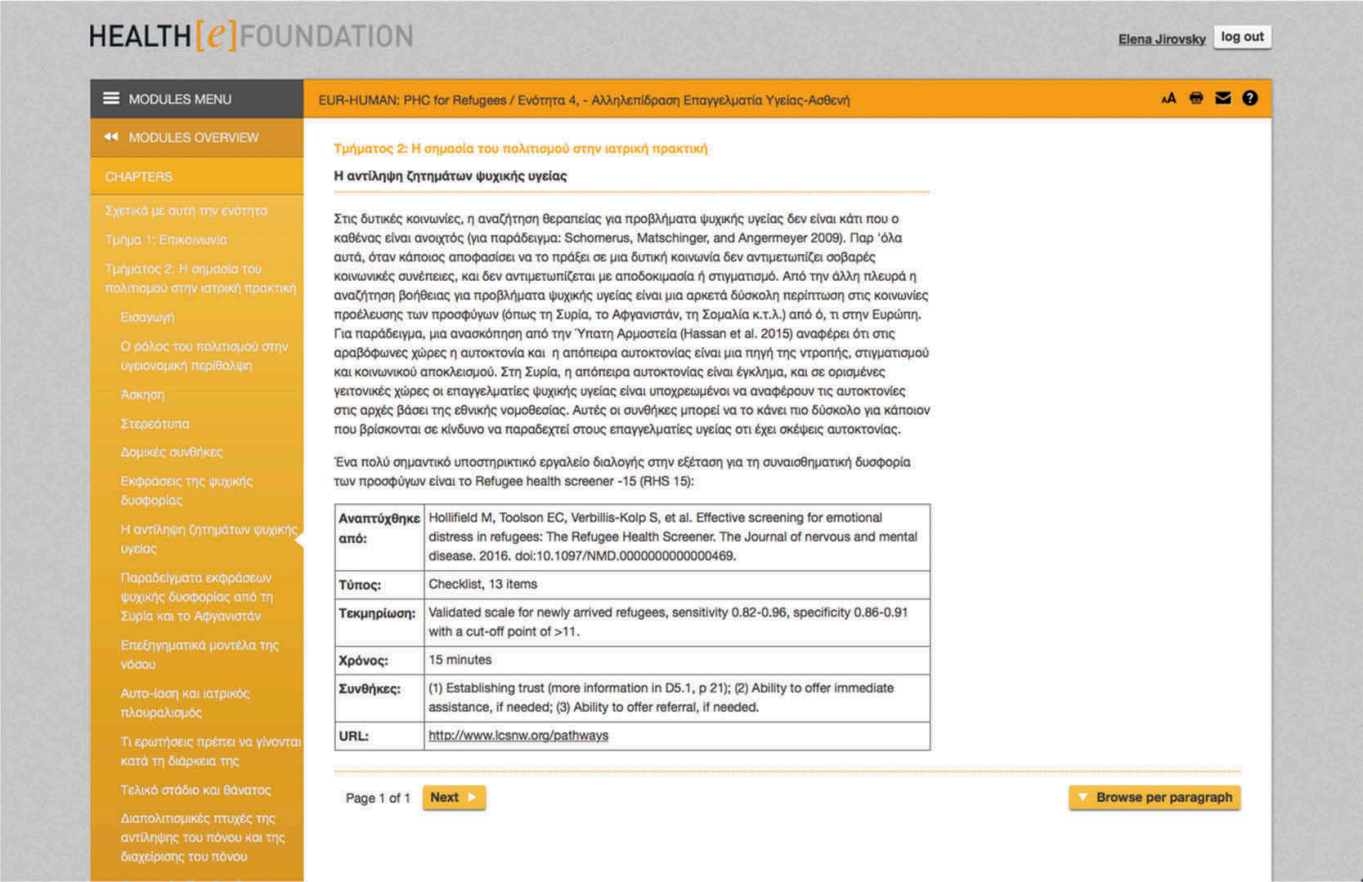
Module 4 – Perception of mental health issues (Greek).

Module 4 is split into two sections. First, it gives an overview on communication principles in health care and issues of intercultural communication. The second part of Module 4 gives an introduction into socioeconomic and cultural aspects of health and illness, distress, or pain. Thus, the module supports PHC professionals in providing culturally sensitive health care for the newly arrived refugee and other migrant population (see Figure 3).

Module 5: Mental health.

Module 5 gives the learner insights in the background and origin of refugees’ mental health problems and the associated risk factors, whilst, at the same time, ensuring adequate information on the specific mental health issue signs a PHC professionals may encounter such as in terms of manifestation of grief, depression and somatic expressions of distress. Furthermore, screening tools and possible treatments for mental health problems, as well as other options for psychosocial support of refugees and other migrants, are introduced.

Module 6: Sexual and reproductive health.

Module 6 introduces the learner to specific issues concerning the sexual and reproductive health of female refugees and other female migrants under difficult living conditions such as in refugee homes. Various topics such as special needs in the peri- and post-natal phase, menstruation, contraception, abortion, and sexually transmitted infections (STI) are discussed. Furthermore, the module informs the learners of red flags and health risks pertaining to sexual and gender-based violence among refugees and other migrants.

Module 7: Child health.

Module 7 is widely based on the Austrian Recommendations of the Working Group for Refugee Children [26]; it addresses probable infectious diseases among refugee children, necessary vaccinations, prevention of physical and mental health issues, as well as how to deal with refugee children in the pediatric practice.

Module 8: Chronic disease, health promotion, and prevention.

Module 8 provides the learners with an overview on possible options for health promotion and approaches to chronic diseases among the refugee and other migrant population. Finally, the module provides a comprehensive list of refugee aid organizations for the psychosocial support of refugees and other migrant groups in the destination.

### Continued medical education (CME) evaluation

In Greece, it was possible to directly observe the application of the newly gained knowledge after the course in a refugee camp on the island Lesvos. However, in Austria, Croatia, Hungary, Italy and Slovenia, the targeted PHC professionals work in their individual practices in different locations. Due to this starting situation, it was clear that in these settings, the online course participants would apply the newly learned knowledge without a possibility for the research team to directly observe the application.

For each module, except for the introductory module, a pre- and a post-test of knowledge was established to assess the progress and the knowledge increase of participants of the EUR-HUMAN online course. The authors of the individual modules prepared multiple choice questions (MCQs) based on the content they developed. There was no minimum score for the knowledge pre-test, however, the minimum score for a passing mark of the knowledge post-test to pass was set at 75%. It was possible to do three attempts. If the third attempt failed for at least one module, the certificate of completion for the whole course was not granted. The pre- and post-tests of knowledge allowed the participants to see their personal knowledge gain on issues regarding the health care for refugees and other migrants. The learning curve can be approximated by comparing the scores of the knowledge pre-test and the post-tests for each module and assessing the increase.

In Austria, the course was accredited by the Austrian Physicians Chamber with 10 Continuous Medical Education (CME) credits. In Croatia, the course was accredited by the Croatian Medical Chamber with 7.5 CME credits. In Slovenia, the course was accredited both by the Slovenian Medical Chamber (24 CME credits), and the Chamber of Nurses (25 CME credits). In Hungary, the online course was accredited by the official portal of the University of Debrecen (OFTEX) with 20 CME points for GPs, occupational specialists, internists, and pediatricians. In Greece and Italy, no CME credits were negotiated; participants only received a certification.

Additionally, as part of WP7 of the EUR-HUMAN project, all participants were invited to participate in an online evaluation survey after the course to assess the acceptability and practicability of the course. A tailored version of the NoMAD questionnaire, based on the Normalization Process Theory (NPT), was used to gather respondents’ views on different aspects of usability, implementation, and integration of the course into primary care services [27].

This paper refers to selected aspects of the NoMAD online survey results and points to findings of the questionnaire regarding the implementation of PHC services, the course experience and the appreciation of the course (see Table 1). A detailed presentation of the methods and findings of the survey conducted using the NoMAD questionnaire will be published in a subsequent paper from the EUR-HUMAN Consortium.

**Table 1.** Average results of pre- and post-test (all countries).

	Average pre-test %	Average post-test %	Difference pre-post-test	Average attempts per module %
Acute health needs of refugees	54	91	36	1,19
Legal issues	71	90	19	1,15
Provider–patient interaction	66	89	23	1,14
Mental health	66	83	17	1,34
Sexual- and reproductive health	77	88	11	1,14
Child health	57	80	23	1,56
Chronic disease, health promotion, and prevention	47	86	39	1,38

For this paper, demographic variables from the NoMAD questionnaire such as gender, completion of the course, profession, practice specialty, average results pre- and post-test, time needed to complete all modules, appreciation, as well as importance for the daily practice were chosen. The survey asked respondents the following questions regarding appreciation: ‘Please indicate what modules of the online training course you studied and that you fully appreciate’ and ‘Please indicate what modules of the online training course you studied and you appreciate less.’ Appreciation was not further defined. The participants could freely enter their profession and practice specialty when appropriate; as some participants did not specify more than that they were working in health care, for the analysis, the variable undefined or other health care workers was created. Another question concerned the willingness to support the training program by promoting; it involved the variables no statement, strongly agree, agree, and neither agree nor disagree. A convenience sampling was used.

### Data analysis

The results of the knowledge tests were collected via a statistical tool, which was directly integrated in the online course by the Health[e]Foundation, with the data transferred to MedUni. The highest possible score for each test was 100%. Points for each knowledge pre- and post-test regarding the different modules are presented in the results’ section, as are the differences between the pre- and post-test per module. The data from the online evaluation survey and the data collected via the pre- and post-test of knowledge was analyzed descriptively.

## Results

A total of 390 participants registered for the online course in 6 different countries with 44.9% (n = 175) completing all modules (Table 2). Among the participants in all countries were 47.7% medical doctors and 58.1% of them were general practitioners. Among all participants, 8.2% were undefined or other health care workers including midwives, infection control specialists, nutritionists, public health specialists. Several migration officers (2.1%) and health managers (1.5%) participated (Table 3). For all course versions except the Arabic, the majority of the participants were female (Table 2).

**Table 2.** Sociodemographic characteristics of CME participants (N = 390)*.

		Gender distribution per country**				
	Number of participants per country***	Female	Male	⊘ age per country **	Participants completing the course***	
Country	N	%	%	years	N	%
Austria German version	65	61%	39%	52	25	38.5
Austria Arabic version	37	24%	76%	35	25	67.6
Greece	17	65%	35%	na	5	29.4
Croatia	36	79%	21%	na	14	38.9
Slovenia	34	80%	20%	na	24	70.6
Hungary	88	39.8%	60.2%	na	16	18.2
Italy	112	na	na	na	66	58.9
**Total**	**390**				**175**	**44.9**

*Totals may not add to 100% due to missing data

**as of 19 December 2016

***as of 3 January 2017

**Table 3.** Profession of the participants***.

Profession of participants (all countries) (N = 390)	Number of Participants	
	N	%
Medical doctors	186	47.7
Participants without specification	130	33.3
Undefined and other health care workers (including midwives, infection control, nutritionists, public health, clinical psychology)	32	8.2
Other occupations and scientists (including veterinary medicine, university lecturers, social media, anthropology)	12	3.1
NGO	9	2.3
Migration officer	8	2.1
Nurse	7	1.8
Health manager	6	1.5
**Total**	**390**	
**Practice specialty medical doctors (N = 186)**		
General Practitioners	108	58.1
Undefined and other MD (including dermatologists, gerontologists, occupational health, dentists, anesthesiologists)	32	17.2
Emergency doctors	10	5.4
Gynecology	8	4.3
Pediatrics	8	4.3
Neurology	7	3.8
Epidemiology	5	2.7
General surgery	4	2.2
Psychiatry	4	2.2
**Total**	**186**	

***as of 3 January 2017

The knowledge pre- and post-test results differed depending on the module (Table 1): While the average results of the pre-test results were relatively low for Modules 2 (acute health needs) and 8 (chronic disease) (54% and 47% respectively), the pre-test results for the modules 3 and 6 were comparably high (71% and 77%). The average pre-test results for module 7 were comparably low with 57 % and the average post-test results remained lower than for the other modules with 80%. Significant pre-post changes were obtained for Module 2, with a difference of 36 points in the average score of the results, and for Module 8, with a difference of 39 points in the average score of the results. The lowest difference was measured in the assessments for Module 6, with 11 points of difference between the averages of knowledge pre- and post-test results. For Modules 5 and 3 the difference between the averages of knowledge pre- and post-test results was slightly higher than for Module 6 with respectively 17 and 19 points. For both Modules 4 and 7, the difference was 23 points.

In total, 123 course participants responded to the invitation for the online evaluation survey and filled out the online survey (Table 4). Their statements show that the mean time for completing the course was 10.77 hours. Module 3 (legal issues) and module 5 (mental health) were the most highly evaluated modules, as 57.7% of the survey participants fully appreciated them. Only 40.7% of the survey participants fully appreciated module 8 (chronic disease health promotion, and prevention). Module 8 was also considered less appreciated by 21.1% of the survey participants while, in comparison, module 2 (acute health needs) was less appreciated only by 4.1% of the survey participants. Above that, 53.7% of participants fully appreciated module 4 (provider–patient interaction). The latter was also found particularly important for daily practice by 52.9% of the Austrian survey participants.

**Table 4.** CME process evaluation.

Modules	Fully appreciated (n = 123)*			Less appreciated (n = 123)*				Particularly important for daily practice (n = 34)**		
	N		%	N		%		N	%	
Acute health needs of refugees	58		47.2	5		4.1		15	44.1	
Legal issues	71		57.7	15		12.2		15	44.1	
Provider–patient interaction	66		53.7	18		14.6		18	52.9	
Mental health	71		57.7	19		15.4		14	41.2	
Sexual- and reproductive health	59		48.0	19		15.4		14	41.2	
Child health	59		48.0	20		16.3		14	41.2	
Chronic disease, health promotion, and prevention	50		40.7	26		21.1		13	38.2	
	Minimum			Maximum		Mean		Standard variance		
Time needed to complete the course in hours*** (N = 123)	1			72		10.77		9.66		
	No statement			Strongly agree		Agree		Neither agree nor disagree		
	N	%		N	%	N	%	N		%
I am willing to support the training program by promoting it (N = 123)	32	26		34	27.6	49	39.8	8		6.5

*Multiple answer options

**Question only part of the evaluation for Austrian Arabic and German version

***In hours

A majority of the survey participants strongly agreed or agreed that they are willing to support the online course by promoting it among their peers.

## Discussion

### Main findings

Research has shown that PHC professionals and refugees or other migrants face equally great challenges when it comes to PHC encounters during the flight, upon and after arrival in the destination countries [15,16,18–20]. In response to these challenges, an online course/CME course for PHC professionals was developed, offering comprehensive knowledge on the issues of refugees’ and other migrants’ health to participants from different countries. Studies show the positive effects of such web-based CME courses [28,29].

Our findings indicate that the participants of our online course were able to expand their knowledge on PHC for refugees and that the course positively influenced the daily practice of PHC providers among the participants, according to their self-assessment. An analysis of the knowledge pre- and post-test differences showed that there were significant changes in the pre- and post-test results of the different modules pointing to a knowledge gain of our participants. We found that in particular modules the knowledge pre-test results were already very high and the knowledge gain therefore comparably lower than for other modules. The participants seem to have been well informed particularly well in the topics *legal issues* (Module 3) and *sexual and reproductive health* (Module 6) prior to the online course. Our findings indicate that the participants had the lowest knowledge on the topic *child health* (Module 7) prior to the online course; the participants’ knowledge also remained comparably low after completion of this module.

The module 4 on provider–patient interaction/intercultural communication was deemed to be particularly important by our participants. This need is consistent with previous reports in the literature focusing on communication [30–33].

We were able to strengthen the target group in providing health care for refugees and other migrants. In general, our CME course was well accepted as indicated by the CME evaluation of our participants.

### Related work

In developing this CME, we found other CME material that covers similar subject areas in regard to migrants or refugees: MEM-TP (http://www.mem-tp.org) and SH-CAPAC (http://www.sh-capac.org). Both of these courses, developed under previous (MEM-TP) and the same as EUR-HUMAN (SH-CAPAC) funding actions of the European Union (CHAFEA) have complementary material to our online course and touch upon similar aspects in terms of minority groups (i.e. Roma for MEM-TP) and other public health needs (SH-CAPAC) in the context of care refugees and migrants to a wide variety of target groups in health care. Both projects have their material available online under the Creative Commons license, and we, therefore, cross-referenced and linked, as appropriate, to the content of those courses in our own course, whenever appropriate.

However, while both abovementioned courses are designed to convey knowledge, the approach of delivery requires they are conducted over an extended time period and several weeks to months are required for their completion, whereas our course aims at rapid capacity increase, conveying much needed knowledge about the treatment of refugees and other migrants in short time and in a flexible and user-friendly manner.

### Strengths and limitations

A strength of the course is that it is concise while it is rich in information. In the development of our course, a key consideration was brevity, to allow a greater number of participants to take part despite limited time due to work realities as PHC professionals, as they usually do not have much time available and especially in PHC [25]. The users can always further investigate on particular topics on their own, but they would still have acquired a sound basis through the knowledge gained in our course. These facts suggest that a concise web-based solution with an approximate time frame of 8–10 hours represents be the best option in terms of feasibility and acceptability. As shown above, participants needed a mean time of 10.77 hours to complete the course.

A main strength of the online format is its online accessibility. We choose an Internet platform since it allows for easy access, flexibility, and updateability. Any web-enabled device at any location can be used. Thus, there is neither the need for travel nor for following a set time schedule of a face-to-face training action. The online format offers the participants the liberty to choose the moment and duration of their study time. They can log in and continue the course whenever their schedule allows it. Furthermore, in the online course, the sequence of the modules is not specified. Hence, the participants can freely navigate between modules and chapters according to their preference and needs.

However, this accessibility and flexibility comes with a prize: there are only limited possibilities for interaction with other course participants. It constitutes a weakness of the course that participants were only minimally able to exchange their ideas or to ask questions. The available interactive options on the course platform were not explicitly highlighted, as the time-restraints and resources of the project did not allow individual tutoring.

It can also be considered a weakness that the liberal time frame can lead the participants to procrastinate and neglect the course.

The didactical methods and instructional design, as well as the format and framework that is used for the course can be improved. Currently, there is a strong emphasis on text-based learning. The course contains (amongst others) pictures, graphs, and links to videos and webpages, however, more audiovisual material could be added. Time-restraints and resources of the project allowed a development of audio-visual material (video presentations) based on the content solely in Greece. Group activities at the beginning of and during the online course, both online with the use of social media, and face-to-face in form of workshops, could further enrich the participants learning experience.

The use of a single group knowledge pre- and post-test evaluation method could also be considered a limitation. The focus of the course was on knowledge transfer and there was neither funding nor opportunity to survey the impact of the course on the PHC professionals’ practice behavior. Therefore, we were unable to conduct a randomized control study (RCT) in this regard.

In the course of the project it was not possible to directly observe the application of the newly gained knowledge after the course  in all countries. It would be an asset to do a direct evaluation of the acquired knowledge and skills also in other countries to further evaluate the quality of the course. Provided that funding is available, such an evaluation will be considered for the future.

### Long-term objective

The long-term objective of the EUR-HUMAN project was the optimization of health care provision for refugees and other migrants. The course can only be an effective instrument contributing to this long-term objective when relevant implementation factors are met. Like any other instrument or tool that is designed to improve health care provision, implementing the web-based capacity building course for primary health care providers evaluated in this article depends on the presence of the right conditions. In this case internet access and sufficient time to follow the course are indispensable. However, if accessing the content of the modules is seen as a first step, the second step is more challenging. The guidance from the modules needs to be applied in practice, in resource contexts that can differ substantially across regions. In order to maximize the future roll out and to increase the chance that the content of the course becomes an integral part of the health care delivery process for refugees, it is necessary to understand relevant implementation factors for recommended interventions, as well as the extent to which those factors are available in the target area of the online training. Flottorp at al [34]. categorized potential ‘determinants of practice’ into seven domains: guideline factors; individual health professional factors; patient factors; professional interactions; incentives and resources; capacity for organizational change; and social, political and legal factors [34–36]. Knowledge about factors like these, that might differ locally and between professional health care disciplines, can increase the potential of the course in optimizing health care provision.

## Conclusion

The EUR-HUMAN online course is a promising contribution to an optimization of health care for refugees and other migrants, combining and integrating insights from many different disciplines and perspectives. The participants of the course found the training useful and their knowledge had grown shortly after the training. The course is a promising learning tool for PHC professionals. When relevant supportive conditions are met locally, it has the potential to empower them in their work with refugees and other migrants. It appears worthwhile to further disseminate the course and to show policy makers that there are free high-quality tools available to train and support PHC professionals in their work with refugees and other migrants.

## Paper context

Research has shown the need to strengthen primary health care (PHC) professionals in the provision of high-quality care for refugees and other migrants. We aimed at building their capacity with a free e-learning course. The course has the potential to empower PHC professionals in this regard. The availability of free high-quality tools to build the capacity of PHC professionals in their work with refugees and other migrants has to be further promoted among policy makers.
